# Predictive Factors of Spontaneous Bacterial Peritonitis Caused by Gram-Positive Bacteria in Patients With Cirrhosis

**DOI:** 10.1097/MD.0000000000003489

**Published:** 2016-04-29

**Authors:** Jung Ho Kim, Yong Duk Jeon, In Young Jung, Mi Young Ahn, Hea Won Ahn, Jin Young Ahn, Nam Su Ku, Sang Hoon Han, Jun Yong Choi, Sang Hoon Ahn, Young Goo Song, Kwang Hyub Han, June Myung Kim

**Affiliations:** From the Department of Internal Medicine (JHK, YDJ, IYJ, MYA, HWA, JYA, NSK, SHH, JYC, SHA, YGS, KHH, JMK); and AIDS Research Institute, Severance Hospital (YDJ, MYA, HWA, JYA, NSK, SHH, JYC, YGS, JMK), Yonsei University College of Medicine, Seoul, Republic of Korea.

## Abstract

Spontaneous bacterial peritonitis (SBP) in patients with cirrhosis is typically caused by gram-negative bacteria. However, the number of SBP cases due to gram-positive bacteria is steadily increasing. To date, little is known about the predictive factors involved in SBP infections.

We performed a retrospective cohort study of patients (>18 years) with SBP due to gram-positive and -negative bacteria who were enrolled from January 2006 to December 2013 at Severance Hospital in Seoul, Korea where the incidences of hepatitis B virus associated chronic liver disease, cirrhosis, and hepatocellular carcinoma are high. Only the 1st SBP episode for each patient within the study period was included in our analysis.

We identified 77 patients with cirrhosis and SBP. Of these, 27 patients (35%) had gram-positive bacterial infections and 50 patients (65%) had gram-negative bacterial infections. Our univariate analysis revealed that an early stage of cirrhosis (*P* = 0.004), lower creatinine level (*P* = 0.011), lower Sequential Organ Failure Assessment (SOFA) score (*P* = 0.001), lower Model for End-Stage Liver Disease score (*P* = 0.005), and use of systemic antibiotics within 30 days before SBP diagnosis (*P* = 0.03) were significantly associated with gram-positive bacterial infections. Our multivariate analysis indicated that the use of systemic antibiotics within 30 days before SBP diagnosis (odds ratio, 3.94; 95% CI, 1.11–13.96; *P* = 0.033) and a lower SOFA score (odds ratio, 0.56; 95% CI, 0.37–0.86; *P* = 0.007) were independent predictive factors of SBP caused by gram-positive bacterial infections in patients with cirrhosis. However, we did not observe a statistically significant difference in the 28-day mortality between the gram-positive and -negative bacterial infection groups (40.7% vs 46.0%, respectively; *P* = 0.407).

In this study, the incidence rate of SBP caused by gram-positive bacteria in patients with cirrhosis was similar to the rates reported in recently published studies. Furthermore, the use of systemic antibiotics within 30 days before SBP diagnosis and a lower SOFA score were significantly associated with SBP caused by gram-positive bacteria in patients with cirrhosis.

## INTRODUCTION

Spontaneous bacterial peritonitis (SBP) is one of the most frequent bacterial infections in patients with cirrhosis and ascites, occurring in 10% to 25% of these patients.^1^ In addition, SBP is associated with high mortality rates (20%–40%) in these patients.^2–4^ SBP in patients with cirrhosis is typically caused by gram-negative bacteria that are part of the intestinal microbial flora (mainly species in the family Enterobacteriaceae).^5–7^ Thus, 3rd-generation cephalosporins are the initial empirical therapy for SBP according to current guidelines.^8,9^ However, in recent years there has been an increase in invasive procedures, antibiotic prophylaxis, and widespread antibiotic use in patients with cirrhosis, which has changed the bacterial spectrum and lead to an increase in SBP by gram-positive bacteria.^10–13^

As a result of the recent changes in the epidemiology of bacteria that cause SBP, the choice of antibiotics for SBP has become a topic of discussion. It is thus important to understand the predictive factors for SBP caused by gram-positive bacteria in patients with cirrhosis. However, to our knowledge, no published studies have focused on this topic.

We performed the present retrospective cohort study to identify the predictive factors for and outcomes of SBP due to gram-positive versus -negative bacterial infections.

## MATERIALS AND METHODS

### Study Design and Population

A retrospective cohort study was conducted to investigate the predictive factors for SBP caused by gram-positive bacteria in patients with cirrhosis. We enrolled 77 patients with cirrhosis and SBP (>18 years) who were hospitalized at the Severance Hospital in Seoul, Korea from 1 January 2006 to 31 December 2013. We only included the 1st episode of SBP for each patient within the study period in our analysis. Patients with a positive culture for highly suspicious skin contaminants (i.e., coagulase-negative *Staphylococci*, *Corynebacterium*, *Propionibacterium*, or *Bacillus* spp) and those with secondary peritonitis were excluded from this study. Secondary peritonitis was considered in patients with the following cases: polymicrobial infection, peritoneal dialysis, indwelling abdominal catheters, and a recent history of abdominal surgery. We reviewed the medical records of the patients and collected the following information: age, sex, cause of cirrhosis, Child–Pugh score, concomitant hepatocellular carcinoma (HCC), history of undergoing transcatheter arterial chemoembolization, initial presenting symptoms, type of isolated bacteria, gastrointestinal bleeding, presentation of septic shock, laboratory results, history of SBP, and use of systemic antibiotics within 30 days before SBP diagnosis.

The study was approved by the Institutional Review Board (IRB) of Yonsei University Health System Clinical Trial Center. Since the study was retrospective and the study subjects were anonymized, the IRB waived the requirement for written consent from the patients.

### Definitions

SBP was defined by the presence of ascitic fluid with a polymorphonuclear leukocyte (PMN) count of >250 cells/mm^3^, and culture-positive SBP implied a positive culture result in ascitic fluid among SBP. During the study period, 204 patients with cirrhosis and SBP were observed, including 127 culture-negative SBP cases. Cirrhosis in all patients was confirmed by clinical, hematological, and biochemical laboratory findings and ultrasound. Chronic kidney disease (CKD) was defined as a glomerular filtration rate of <60 mL/min/1.73 m^2^ for ≥3 months irrespective of other signs of kidney damage.^14^ Nosocomial infection was defined as an infection that occurred >48 hours after admission to the hospital. Infections diagnosed within the 1st 48 hours of hospitalization were classified as community-acquired infections.^12^ Hepatic encephalopathy was defined as an episode of mental confusion, disorientation, excitation, abnormal behavior, or asterixis.^15^ Gastrointestinal variceal bleeding was confirmed by endoscopy.^16^ Antibiotic prophylaxis therapy was defined as the use of rifaximin (Normix).^17^ Septic shock was defined as sepsis associated with evidence of organ hypoperfusion and a systolic blood pressure of <90 mm Hg, >30 mm Hg less than baseline, or a requirement for the use of a vasopressor to maintain blood pressure.^2^ Clinical manifestations included the presence of the following initial symptoms: fever (temperature of >38 °C), abdominal pain, and diarrhea (defined as >3 loose stools per day).

### Laboratory Tests

Ascitic fluid specimens were obtained aseptically by paracentesis and inoculated into blood culture bottles at the bedside. Ascitic fluid samples were also sent to the microbiology laboratory for PMN counting and Gram staining. We injected 10 mL of ascitic fluid samples into blood bottles (bioMerieux) and cultured using BacT/ALERT 3D system (bioMerieux). Remaining ascitic fluid from each sample was cultured using conventional methods (i.e., MacConkey agar, inoculating blood agar, thioglycollate broth, and phenylethanol agar). After day 3 of incubation and a lack of growth, conventional agar was considered negative and discarded.

### Statistical Analysis

Independent *t* tests were used to compare continuous variables, and Chi-square or Fisher exact tests were used to compare categorical variables. Predictive factors for gram-positive bacterial infections were determined by a multivariate binary logistic regression analysis including significant univariate predictors (*P* < 0.05) using a stepwise backward elimination from the total cohort of culture-positive cases of SBP. *P*-values of <0.05 were considered statistically significant. PASW ver. 18 for Windows (SPSS Inc., Chicago, IL) was used for analyses.

## RESULTS

### Demographic Characteristics

In total, 77 patients with cirrhosis and SBP were identified (61 male and 16 female). Table 1 shows the demographic and clinical characteristics of the patients. The most frequent causes of cirrhosis were hepatitis B virus (55.8%), hepatitis C virus (20.8%), and excessive alcohol ingestion (14.3%). There were 3 patients (3.9%) in Child–Pugh class A, 23 patients (29.9%) in class B, and 51 (66.2%) in class C.

**TABLE 1:**
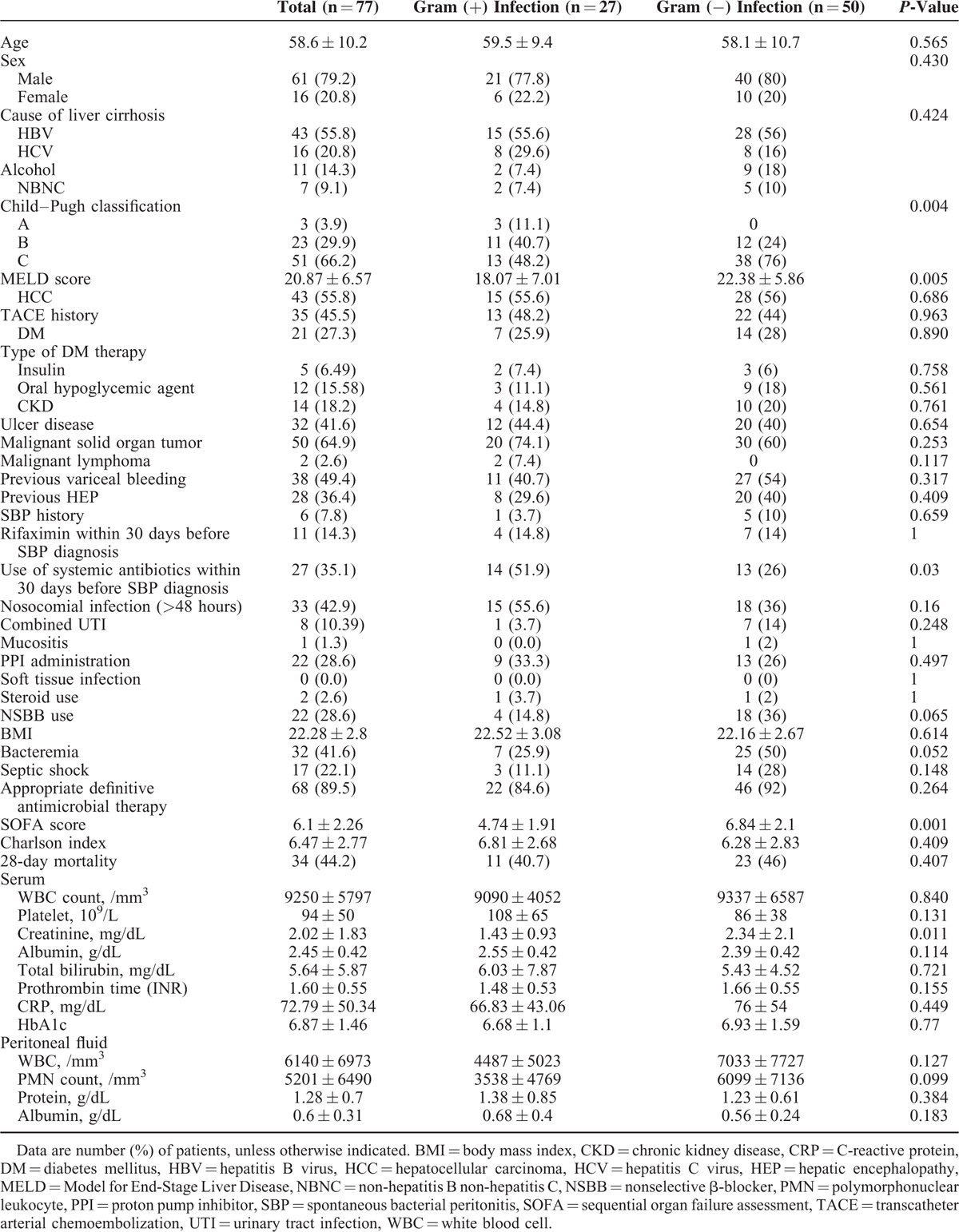
Clinical Characteristics and Laboratory Findings of Patients With Spontaneous Bacterial Peritonitis

### Microbiological Characteristics

The organisms isolated from the ascitic fluid of patients with SBP are listed in Table 2. There were 50 patients (64.9%) with gram-negative infections and 27 patients (35.1%) with gram-positive infections. *Escherichia coli* was the most common isolate (32.5%), followed by *Klebsiella pneumoniae* (19.5%). Eleven of the gram-negative bacteria (22%) were extended spectrum beta-lactamase (ESBL) positive (+). For patients with gram-positive bacterial infections, *Enterococcus* spp and *Staphylococcus aureus* were the most common isolates (13.0%), followed by *Streptococcus* spp (9.1%). *Enterococci* species included 7 *Enterococcus faecium*, 2 *E raffinosus*, and 1 *E faecalis*. Two of the *E faecium* isolates were vancomycin resistant. *Staphylococcus aureus* comprised 6 methicillin-resistant *S aureus* (MRSA) and 4 methicillin-susceptible *S aureus* (MSSA). Antibiotics used for the definitive treatment of SBP were listed in Table 3. We did not observe a statistically significant difference in the proportion of patients who received appropriate definitive antimicrobial therapy considering their susceptibility testing results between the gram-positive and -negative bacterial infection groups (84.6% vs 92%, respectively; *P* = 0.264).

**TABLE 2:**
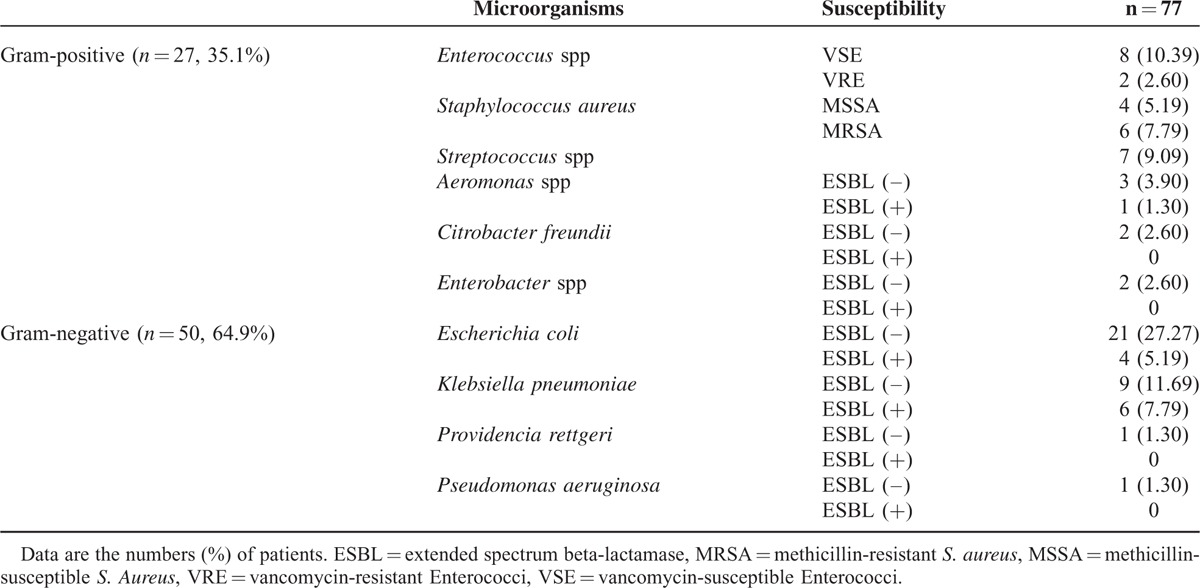
Bacteria Isolated From Ascitic Fluid in Patients With Spontaneous Bacterial Peritonitis

**TABLE 3:**
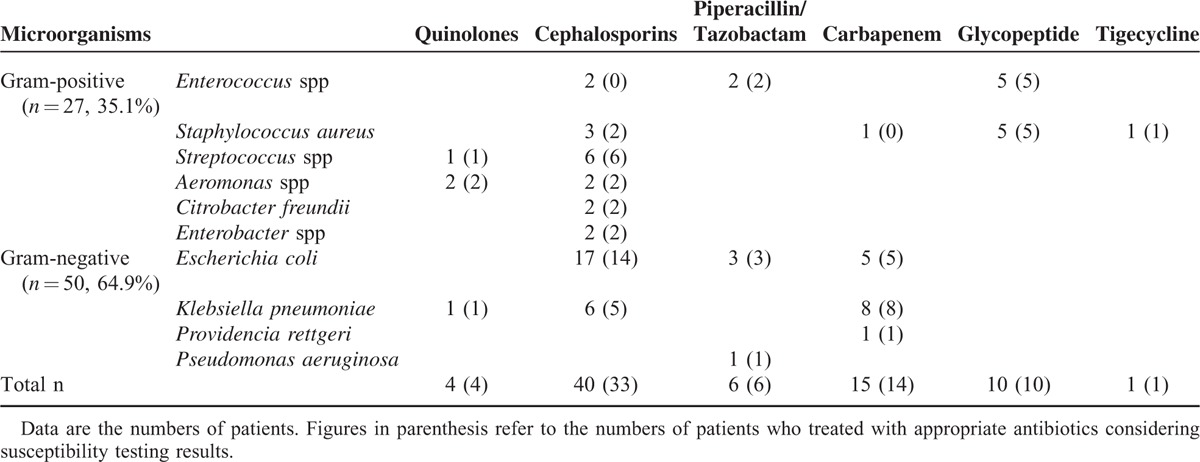
Antibiotics Used for the Definitive Treatment of Spontaneous Bacterial Peritonitis (SBP)

### Predictive Factors of Gram-Positive Bacterial Infections

There were no statistically significant differences in age, sex, cause of cirrhosis, concomitant HCC, history of undergoing transcatheter arterial chemoembolization, underlying diabetes mellitus, CKD, or history of SBP between patients with gram-positive and -negative bacterial infections.

However, the results of our univariate analysis indicated that the following factors were significantly associated with gram-positive bacterial infections: early stage of cirrhosis (*P* = 0.004), a low creatinine level (*P* = 0.011), a low Sequential Organ Failure Assessment (SOFA) score (*P* = 0.001), a low Model for End-Stage Liver Disease score (*P* = 0.005), and use of systemic antibiotics within 30 days before SBP diagnosis (*P* = 0.03). The results of the multivariate analysis revealed that the use of systemic antibiotics within 30 days before SBP diagnosis (odds ratio, 3.94; 95% confidence interval, 1.11–13.96; *P* = 0.033) and a lower SOFA score (odds ratio, 0.56; 95% confidence interval, 0.37–0.86; *P* = 0.007) were independent predictive factors for SBP caused by gram-positive bacterial infections in patients with cirrhosis. However, we did not observe a statistically significant difference in 28-day mortality between the gram-positive and -negative bacterial infection groups (40.7% vs 46.0%, respectively; *P* = 0.407) (Table 4).

**TABLE 4:**
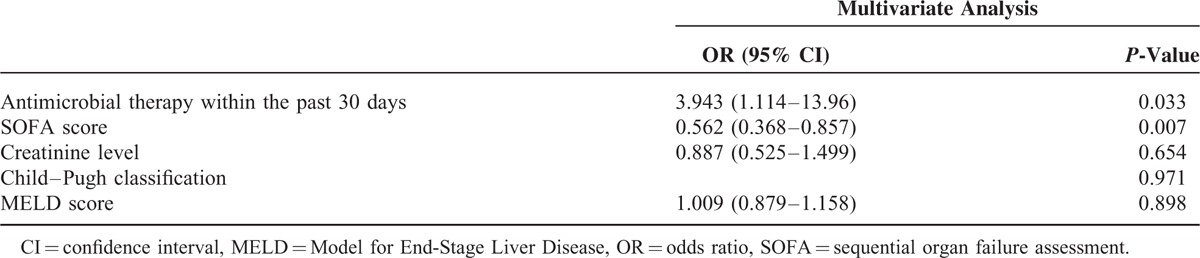
Predictive Factors for Gram-Positive Bacterial Infection in Patients With Spontaneous Bacterial Peritonitis

## DISCUSSION

SBP is a common complication in patients with cirrhosis and ascites despite recent improvements in therapeutic approaches.^1^ Gram-negative bacteria (most frequently *E coli*) through translocation from the intestinal lumen are responsible for the majority of SBP cases.^6^ However, the preponderance of infections caused by gram-negative bacteria due to epidemiological changes has shifted to a higher prevalence of infections being caused by gram-positive cocci.^18^ In a study by Alexopoulou et al,^13^ the majority of isolated pathogens from patients with SBP were gram-positive cocci (55%). Our findings are in agreement with several recent reports that showed a high frequency of gram-positive bacterial infections associated with SBP.^11,13,19^

We observed that the use of systemic antibiotics within 30 days before SBP diagnosis and a lower SOFA score were independent predictors of SBP caused by gram-positive bacteria in patients with cirrhosis. However, the effects of proton pump inhibitor administration, taking nonselective β-blocker and taking rifaximin for prophylaxis within 30 days before SBP diagnosis on the predominance of gram-positive bacteria could not be proven in our study.

In this study, the use of systemic antibiotics within 30 days before SBP diagnosis was an independent predictive factor in patients with cirrhosis and SBP caused by gram-positive bacterial infections. Innate and adaptive immune dysfunction, also referred to as cirrhosis-associated immune dysfunction syndrome, is a major component of cirrhosis.^20^ Bacterial infections are common and represent important causes of liver-related complications, progression of liver failure, and mortality related to cirrhosis.^21^ In actual fact, bacterial infections may trigger the development of gastrointestinal bleeding, hepatic encephalopathy, hypervolemic hyponatremia, kidney disease, and acute-on-chronic liver failure.^21,22^ Thus, early diagnosis and prompt initiation of adequate antibiotic therapy is essential in the management of patients with cirrhosis and bacterial infections.^23^ As a result, patients with cirrhosis consume more antibiotics than the general population. In our study, 27 patients (35.1%) had taken systemic antimicrobial therapy within the past 30 days, but in cases of gram-positive bacterial infections, the proportion increased to 51.9% (14 of 27). This result might be attributed to 2 factors. First, although there were no statistically significant differences between the 2 groups in the rate of nosocomial infections, gram-positive bacterial infections were reported more frequently in the hospital than gram-negative infections (55.6% vs 36.0%, respectively; *P* = 0.16). Second, the prevalence of infections caused by multiresistant bacteria (e.g., methicillin-resistant *S aureus* and *E faecium*) is increasing in cirrhosis patients.^19^

In addition, this study showed that a lower SOFA score was associated with SBP caused by gram-positive bacterial infections in patients with cirrhosis. The SOFA score is a simple but effective method to describe organ dysfunction/failure in critically ill patients not only in the ICU but also in other places, such as emergency departments.^24,25^ As many patients with SBP were admitted via the emergency department, calculating the SOFA score is important for predicting the outcome along with other clinical information. Regular, repeated scoring enables a patient's condition and disease development to be monitored and better understood.^26^ In our study, gram-negative bacterial infections were associated with a higher SOFA score than gram-positive infections (6.84 vs 4.74, respectively; *P* = 0.005). This result is likely attributed to the higher incidence of gram-negative bacterial infections in advanced cirrhosis. According to the most widely accepted theory, the initial event of SBP is translocation of intestinal bacteria to the mesenteric lymph nodes and subsequently into the systemic circulation. In patients with impaired reticuloendothelial phagocytic activity, this process becomes clinically relevant and leads to spontaneous bacteremia. When defense mechanisms within ascitic fluid are reduced, the circulating organisms colonize the peritoneal fluid, leading to SBP.^10^ Thus, one of the steps in SBP was a disturbance in gut flora caused by commonly overgrowing gram-negative aerobic bacteria, such as *E coli*. Bacteria within the gut lumen can traverse the intestinal wall, colonize mesenteric lymph nodes, move into the systemic circulation, and enter the ascitic fluid through the liver.^13^ As a result, if cirrhosis progresses, the chance of obtaining SBP by gram-negative bacteria increases. This could be one of the reasons why gram-negative bacterial infections were associated with a higher SOFA score in the present study.

This study had several limitations that should be addressed. First, the patients included in this study were from a single center in the Republic of Korea, where the incidence of hepatitis B virus-associated CKD, cirrhosis, and HCC are high. Therefore, the results might not be applicable to different hospitals. Second, there is potential for bias and inaccurate data collection because it was a retrospective study. Further prospective studies conducted in larger patient populations involving multiple centers will be necessary. Third, this study did not include patients with bacterial ascites (defined as a positive culture result in ascitic fluid without an elevated PMN count). In clinical practice, patients with this condition are occasionally treated with antibiotics. However, in this study, we just included patients with culture-positive SBP; thus, selection bias might have occurred.

## CONCLUSIONS

In conclusion, our findings are in agreement with several recent studies that reported a high frequency of gram-positive SBP.^11,13,19^ In addition, the use of systemic antibiotics within 30 days before SBP diagnosis and a lower SOFA score were significantly associated with SBP caused by gram-positive bacteria in patients with cirrhosis. Therefore, it is important that physicians take all of these factors into account when diagnosing and treating patients with cirrhosis and SBP.
